# Refined separation of combined Fe–Hf from rock matrices for isotope analyses using AG-MP-1M and Ln-Spec chromatographic extraction resins

**DOI:** 10.1016/j.mex.2014.08.004

**Published:** 2014-08-28

**Authors:** Ting Cheng, Oliver Nebel, Paolo A. Sossi, Fukun Chen

**Affiliations:** a Chinese Academy of Sciences, Key Laboratory of Crust-Mantle Materials and Environments, School of Earth and Space Sciences, University of Science and Technology of China, Hefei 230026, China; b Research School of Earth Sciences, The Australian National University, 0200 Acton, ACT, Australia

**Keywords:** Fe–Hf separation, AG-MP-1M resin, Ln-Spec resin

## Abstract

A combined procedure for separating Fe and Hf from a single rock digestion is presented. In a two-stage chromatographic extraction process, a purified Fe fraction is first quantitatively separated from the rock matrix using AG-MP-1M resin in HCl. Hafnium is subsequently isolated using a modified version of a commonly applied method using Eichrom LN-Spec resin. Our combined method includes:

• Purification of Fe from the rock matrix using HCl, ready for mass spectrometric analysis.
• Direct loading of the matrix onto the resin that is used for Hf purification.
• Collection of a Fe-free Hf fraction.

## Graphical abstract

**Figure:**
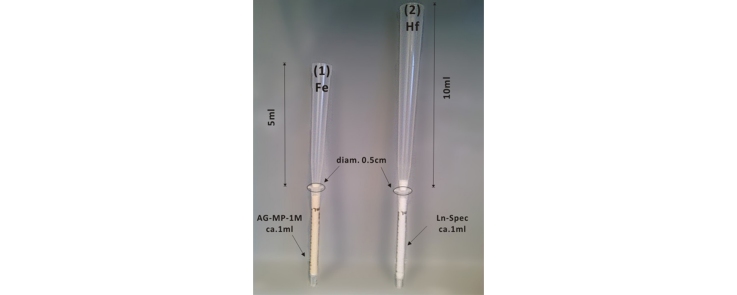

## Method details

We combined two individual separation techniques to purify Fe and Hf from rock matrices for subsequent isotope analyses using mass spectrometry from a single dissolution batch. Iron is particularly problematic due to its incomplete separation from Hf, which can cause isobaric interferences (matrix effects) during Hf isotope mass-spectrometric measurements [1–4]. We present a combined and refined version of a “clean-up” step initially designed to remove Fe that is required for Hf purification. Using this method facilitates the collection and subsequent analyses of both elements from a single rock matrix.

The procedure follows the steps:

1. Rock powder is dissolved in a concentrated acid mixture of 24 M HF–15.7 M HNO_3_ in a ratio of 1:2.
2. Sample treatment with concentrated HNO_3_ after evaporation breaks down fluoride (as e.g., CaF) bonds that form during the chemical dissolution.
3. Final uptake in strong (9 M) HCl ensures the full dissolution of sample material and the formation of (iron) chloro-compounds.
4. Samples are first passed through Bio-Rad AG-MP-1M cation resin in steps of 9-5-1M HCl, separating Fe (1 M) from the rock matrix (9 + 5 M)
5. Subsequently Hf (and possibly Lu for old samples that require age correction) is separated from the 9 M HCl matrix fraction (after diluting it) using Eichrom Ln-Spec.

## Method recipe

### Rock dissolution procedure

All acids used during the purification procedure (hydrofluoric, hydrochloric, nitric acid) have been purified by sub-boiling distillation in Teflon (Hf) or quarz (HCl, HNO_3_) distils. Rock powder is dissolved in 5 mL Teflon vials in a 1:2 acid mixture of 2–3 mL 15.7 M HNO_3_ + 24 M HF. Dissolution is usually performed at ca. 150 °C on a hotplate for 24 h. Samples that contain zircon (as e.g., granites) or chromite (e.g., dunites) require dissolution in steel-jacketed Teflon containers (bombs), in an oven at ca. 200 °C for 48 h to ensure the breakdown of all mineral phases. After evaporation of the dissolving acid, we added several drops of concentrate nitric acid and one drop of 0.5 M HCl + 0.5 M HF mixed acid to each sample to break down CaF bonds and achieve total dissolution and repeated treatment may be necessary. After a final uptake in 9 M HCl and overnight storage of the vials on the hotplate, we could not observe any macroscopic solid residues in the beakers. Samples equilibrated in 9 M HCl should be centrifuged to fully ensure that no microscopic residues are loaded onto the column, and can subsequently be pipetted onto the chemical separation resin.

### Iron purification

**Fig. 1:**
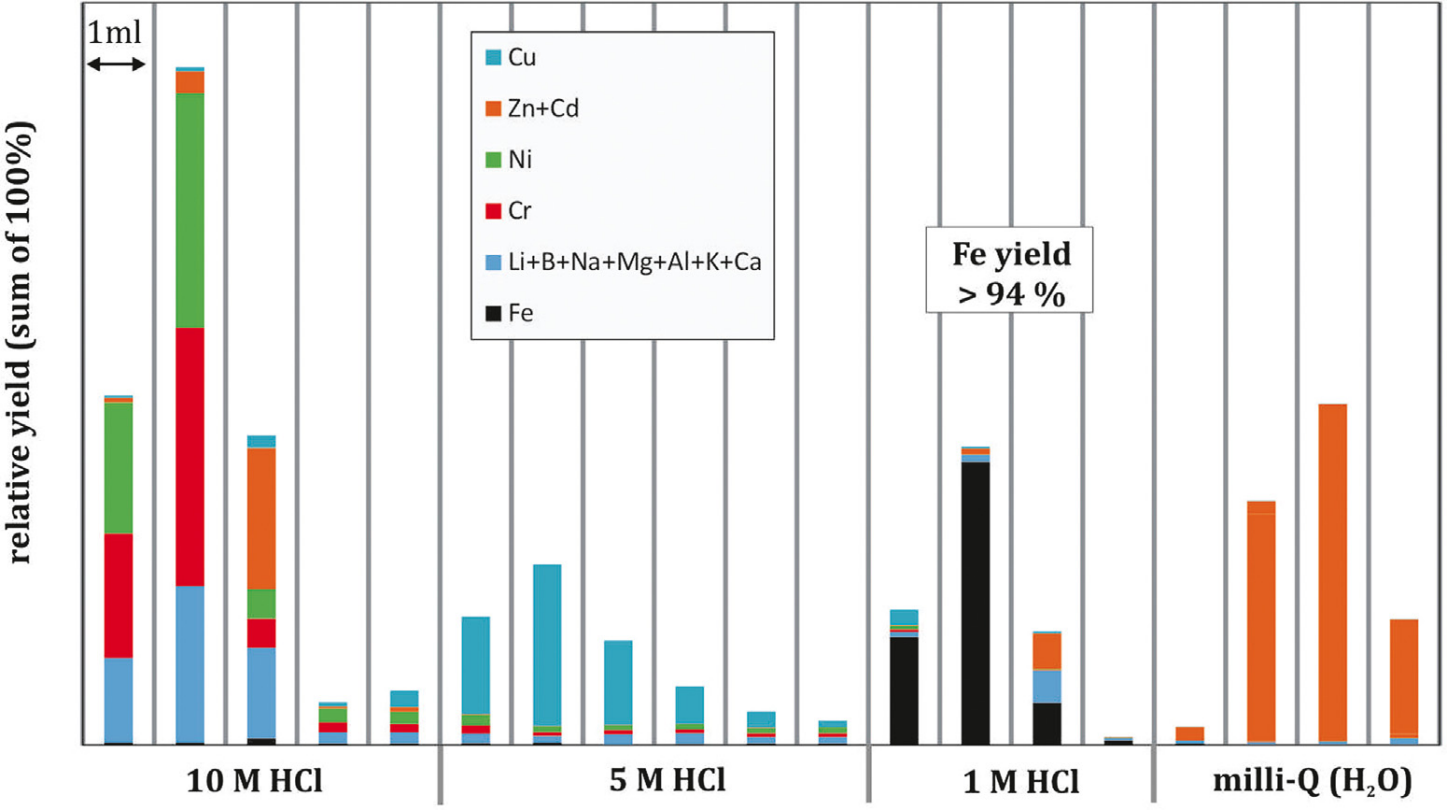
Column extraction scheme for various metals (experiment 1), including Fe, as obtained by ICP-OES, demonstrating the effectiveness of the single Fe column separation technique (AG-MP-1M resin).

For stable isotope analyses and without a double-spike, it is essential that column yields are 100%, because of possible mass-dependent isotope fractionation during the progressive elution of Fe from the chromatographic resin [5–7]. A second prerequisite is the total separation of Fe from Hf, because Fe may cause polymeric interferences and induce matrix effects during Hf isotope measurements. Ideally, it is further desirable to know when other lithophile elements that are frequently used for isotope analyses are eluted from the columns, namely, the parent nuclide Lu, which is required for age correction purposes when analyzing Hf. We performed a simple test by doping the Fe column with various elements (at ca. 10 ppm concentration for each element) and followed the standard elution procedure. In a first experiment, we tested the elution of Fe to ensure no Fe was lost during the separation procedure (Fig. 1), which could result in mass dependent isotope fractionation. In a subsequent test, in agreement with partition coefficients for high field-strength elements (HFSE), rare earth elements (REE) and alkaline earth elements on cation resin, these elements were eluted in strong HCl in the first 9 M elution step.

For rock powders, dissolved samples are taken up in 9 M HCl and centrifuged to remove possible residues. The solution is then transferred using a pipette onto pre-conditioned columns filled with 1 mL AG-MP-1M resin. Columns were prepared from modified polyethylene one-time syringes with a length of ca. 10 cm and an internal column diameter of 0.5 cm, equipped with a frit at the bottom and topped with a 5-mL one-time pipette tip that acts as an acid reservoir. This material proved to be acid resistant, easy to clean with no detectable blanks, and cheap. Hafnium, Lu and other matrix elements were eluted as anionic chloric-complexes with the 9 M HCl acid. We collected this fraction in 30 mL PFA beakers for further purification of Hf and Lu, if required. After rinsing the column with 5 M HCl and eluting further semi and transitional metals (see Fig. 1), the Fe fraction is then eluted with 1 M HCl, subsequently evaporated to dryness, and is ready for isotope analyses. The 9 M HCl fraction, upon dilution, can be directly transferred to the Hf columns.

### Hafnium purification

The separation of Lu and Hf from the rock matrix is modified from the method described in [8]. The 9 M HCl matrix fraction collected from the Fe-separation columns contains Lu and Hf. The solution can be diluted to 6 M HCl (if only Hf is required) or 3 M HCl, if Lu and Hf is required, and then directly loaded onto column B (1 mL LN-spec resin). The amount of acid should not compromise the elution scheme, as HFSE (in 6 M HCl) or heavy REE (in 3 M HCL) should be filtered by the resin from the solution. Evaporation to dryness and subsequent uptake in the respective acids is also possible if smaller acids loads are desirable, as e.g., for the collection of Sm, Nd, Rb and Sr, which are directly eluted with the initial column load. Titanium elution follows the recipe of Münker et al. [8] using a 0.09 M Hcit + 0.4 M HNO_3_ + 1% H_2_O_2_ acid mixture. Finally we elute Hf (+Zr) with 0.5 M HF, gently evaporate them to dryness, and transferred them to tubes in a 1 mL 0.54 M HNO_3_ + 0.24 M HF mixed acid.

**Fig. 2:**
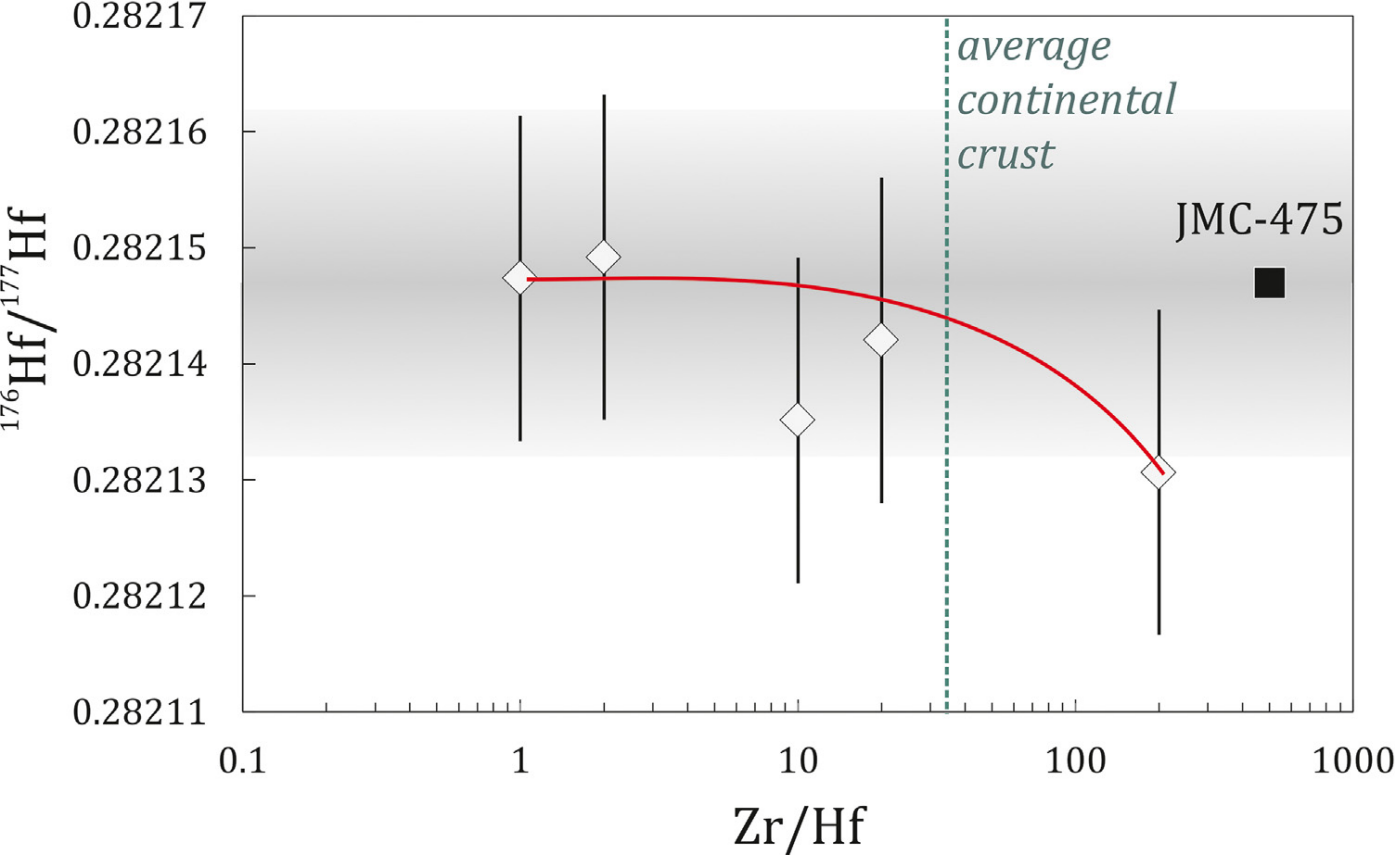
Relationship between ^176^Hf/^177^Hf and Zr/Hf ratios for JMC-45 with Zr.

A modification to the original chemistry by Münker et al. [8] is the combined elution of Zr and Hf. To test if Zr has an effect on Hf measurement, we added a Zr solution to the Hf cut and find that only with extreme Zr/Hf, exceeding those of natural rocks, Zr can alter the Hf isotope composition outside analytical uncertainty. These findings are in agreement with previous studies on a different instrument that performed similar tests and concluded that it is not necessary to separate Hf from Zr for analysis by MC-ICP-MS. Goolaerts et al. [9] carried out a set of experiments on a double-focusing Nu015 MC-ICP-MS and indicated that Zr/Hf up to 800 have no effect on Hf isotopic composition and only a moderate effect on the precision, in contrast to thermal ionization mass spectrometry (TIMS). Qi Chang-shi et al. [10] also used a similar experiment on a single-focusing Micromass Isoprobe MC-ICP-MS to demonstrate that Zr/Hf ≤ 120 show no effect on the Hf measurements. In our test, we used five JMC-475 solutions (50 ppb), each doped with different concentrations of a Zr ICP standard solution (50–100–500–1,000–10,000 ppb). Results are illustrated in Fig. 2, showing that all analyses plot within uncertainty in the range of the average JMC-475 solution of the analytical session. Although the solution with Zr/Hf = 200 shows a drift towards lower Hf isotope compositions, it is not resolvable outside our external reproducibility of ±0.000014 on the ^176^Hf/^177^Hf (Table 1).

### Data assurance

Isotope analyses using MC-ICP-MS follow standard procedures described in [7] for Fe, and Nebel et al. [11] for Hf. Both of those studies list a number of standard reference materials (SRM) that ensure that our measurement protocols are rigorous and reproducible. For this particular study, we tested our method with analyses for SRM BHVO-2 and BCR-2. We used approximately 50 mg aliquots of BHVO-2 and BCR-2 and applied the procedure described above. Hafnium isotope results for BHVO-2 and BCR-2 are ^176^Hf/^177^Hf = 0.283109 ± 02 and 0.282869 ± 03, respectively which are in good agreement with previously reported value (cf. Table 2). Corresponding Fe isotope analyzed yield values of δ^5^^7^Fe = 0.20 ± 0.02 (1 s.d.) and 0.16 ± 0.01 (1 s.d.) relative to the IRMM-014 standard solution for BHVO-2 and BCR-2, respectively. Again both values are identical to recommended values (Table 2). The total procedural blanks measured for Hf were 25 pg, blanks for Fe were negligible as to the large amount of Fe in the sample.

Overall, we find that the presented technique is applicable to mafic rock matrices, and can likely be applied to a large variety of rocks and minerals of different composition and matrices. Limitations are high Fe/Hf of samples, so that application to, for example, magnetite, may need adjustment.

### Tips and tricks

1. In the process of sample dissolution, samples should be dissolved in strong HCl after the treatment with concentrated HNO_3_ for the breakdown of CaF. Even if residues remain after the aforementioned step, they should dissolve in this acid and the solution should be a transparent yellow colour. 9 M HCl is used as it is the acid used for first step of the separation technique.
2. Column dimensions used here require ca. 1 mL of AG-MP-1M resin and 1 mL of Ln-resin. The capacity of the AG-MP-1M resin is close to its maximum for 100 mg of sample material and ca. 5–7 wt.% Fe. For very Fe rich samples or more sample material (sometimes required for Hf isotope analyses), we recommend repeating the Fe column step. If Fe concentrations are unknown, a simple test for overloading the columns is the colour of the first eluant in 9 M HCl, which should never be yellow (indicating that Fe was eluted from the column).
3. In order to achieve efficient separation of Ti and Hf, solutions containing H_2_O_2_ need to be prepared daily. Ti-complexes form a yellow colour and elution should be continued until the eluant appears colourless.
4. Noteworthy is that using 0.5 M HF to elute Hf will result in a combined HFSE batch, including W. Whereas this does not affect Hf isotope measurements, it may prove problematic for groups using a Hf-180 enriched isotope tracer, because of isobaric interferences of W-180 on Hf-180.

## Additional information

### Background

Since the introduction of solution-based multi-collector mass spectrometers with an inductively coupled plasma source (MC-ICP-MS), Hf isotopes have become a very popular tool in geochemical studies for tracing crustal evolution, mantle geochemistry or in geochronology. The isotope Hf-176 is the decay product of Lu-176 by beta-decay with a half-life of approximately 3.5 ± 0.2 billion years, which makes it applicable for studies ranging from solar system formation to the present day. ^176^Hf/^177^Hf in terrestrial rocks vary by ca. ±0.2%, but they are often much more subtle within single rock suites, necessitating extremely precise analyses. Previous studies have demonstrated that high-precision Hf isotope analyses, commonly reproducible to ±0.005%, require purification of Hf from the rock or mineral matrix to reduce matrix effects in the plasma to insignificant levels. It was further demonstrated that especially the major elements Fe and Ti can seriously affect the isotope analyses of the trace element Hf. The pioneering study by Münker et al. [8] presented a very efficient and rigorous method to eliminate Ti interferences, noting that separation of high-field strength elements with similar radius or charge from each other can prove difficult. The separation from Fe, however, has been an obstacle ever since. This was originally overcome by converting all Fe in solution to Fe^2+^ by the addition of a reducing agent, in this case ascorbic acid. Iron in its reduced Fe^2+^ species has partition coefficients on Ln-Spec resin in the loading acid (3–6 M HCl) of close to zero, similar to other 2+ cations such as Mg^2+^ or Ca^2+^. However, for many geochemical studies, additional information from a single rock or mineral dissolution is desirable, as for example Sr or Nd isotope analyses, which requires additional purification of these elements from the matrix. This is where this method runs short: strong oxidation of ascorbic acid is required during evaporation to avoid organic residues that complicate mass spectrometry. This method is time-consuming, may introduce additional blanks and the oxidation procedure can result in strong reactions that may lead to loss or cross-contamination of samples by ejecting material. In more recent years, stable Fe isotope analyses have further been proven useful for high-temperature rocks, also in combination with Hf isotope analyses [17]. Here we present a simple but efficient method to address both problems: purification of Hf from Fe and collection of a purified Fe species readily available for isotope analyses. To achieve this, we combine two established methods into a single procedure to be more time and cost efficient. We finally test our results with two international standard reference materials.

## Figures and Tables

**Table 1:** Two-column procedure for separation of Fe–Hf.

Step	Column volumes	Acid
Column A (AG-MP-1M 1 mL)		
Pre-cleaning procedure		
Preconditioning	5 mL	9 M HCl
Loading sample (collected for Hf)	1–5 mL	9 M HCl
Eluting matrix (collected for Hf)	5 mL	9 M HCl
Eluting matrix	5 mL	5 M HCl
Collecting Fe	5 mL	1 M HCl
Column B (LN Spec 1 mL)		
Pre-cleaning procedure		
Preconditioning	10 mL	3 M HCl
Loading sample	1–10 mL	3 M HCl
Eluting matrix (Sr) + MREE	10 mL	3 M HCl
Collecting Lu	10 mL	6 M HCl
Rinse (extra wash for high Lu/Hf)	10 mL or more	6 M HCl
Rinse	2 mL + 2 mL	H_2_O
Eluting Ti (when TiO_2_–H_2_O_2_ complex disappears)	∼40 mL	0.09 M Hcit/0.4 M HNO_3_/1%H_2_O_2_
Rinse	2 mL + 2 mL	H_2_O
Collecting Hf (Zr)	10 mL	0.5 M HF

**Table 2:** Comparison of ^176^Hf/^177^Hf, ^56^Fe and δ^57^Fe isotopic analyses results of SRMS with previously recommended value.

SRM	^176^Hf/^177^Hf	
	This study (±2*σ*)	Recommended value
BHVO-2	0.283109 (02)	0.283116 [12] 0.283102 [13] 0.283105 [14] 0.283106 [15]

BCR-2	0.282869 (03)	0.282869 [3] 0.282862 [16] 0.2828670 [14]

SRM	δ^56^Fe	±1 s.d.	δ^57^Fe	±1 s.d
BHVO-2	0.139	0.01	0.202	0.016
BCR-2	0.111	0.004	0.159	0.006

[12] – Bizzarro et al. (2003); [13] – Witting et al. (2006); [14] – Weis et al. (2007); [15] – XH Li et al. (2007); – [3] Ulfbeck et al. (2003); [16] – Vervoort et al. (2004); Literature values for δ^57^Fe are BHVO-2 = 0.18 ± 0.03 and BCR-2 = 0.13 ± 0.02 [7].
